# Degradation of Plastics in Simulated Landfill Conditions

**DOI:** 10.3390/polym13071014

**Published:** 2021-03-25

**Authors:** Quecholac-Piña Xochitl, Hernández-Berriel María del Consuelo, Mañón-Salas María del Consuelo, Espinosa-Valdemar Rosa María, Vázquez-Morillas Alethia

**Affiliations:** 1 Tecnológico Nacional de México/Instituto Tecnológico de Toluca, Av. Tecnológico S/N Col. Agrícola Bellavista, Metepec, Edo de México C.P. 52149, Mexico; xquecholacp@toluca.tecnm.mx (Q.-P.X.); mherdandezb@toluca.tecnm.mx (H.-B.M.d.C.); 2 Sociedad Mexicana de Ciencia y Tecnología Aplicada a Residuos Sólidos, A. C., Priv. Molcajete 44 Fracc. Hacienda de las Fuentes, Calimaya C.P. 52227, Mexico; consuelomanon@gmail.com; 3 Departamento de Energía, Unidad Azcapotzalco, Universidad Autónoma Metropolitana, Av. San Pablo 180, Col. Reynosa Tamaulipas, Azcapotzalco, Ciudad de México C.P. 02200, Mexico; rmev@azc.uam.mx

**Keywords:** oxo-degradable, polyethylene, biodegradation

## Abstract

Different degradable plastics have been promoted as a solution for the accumulation of waste in landfills and the natural environment; in Mexico, the most popular options are oxo-degradable, which degrade in a sequential abiotic–biotic process, and compostable plastics. In this research, high-density polyethylene, oxo-degradable high-density polyethylene, and certified compostable plastic were exposed to simulated landfill conditions in an 854-day-long experiment to assess their degradation. High-density polyethylene showed limited degradation, due mainly to surface erosion, evidenced by a 13% decrease in elongation at break. The pro-oxidant additive in the oxo-degradable plastic increased this loss of mechanical properties to 27%. However, both plastic films kept their physical integrity and high molecular weight by the end of the experiment, evidencing degradation but no biodegradation. While the compostable film fragmented, had a lower molecular weight at the end of the experiment, and decreased the presence of C=O bonds, this degradation took place remarkably slower than expected from a composting process. Results show that oxo-degradable and compostable plastics will not biodegrade readily in landfills. This fact should be known and understood for decision-makers to match the characteristics of the materials to the features of the waste management systems.

## 1. Introduction

Plastics emerged as a basic material for the functioning of the global society in the 20th century. The plastic production has increased continuously since the 1950s, reaching 359 Mt in 2018 [1]. Although they are used in different sectors, such as communication, medicine, agriculture, and transportation, 36% of the global plastic production in 2015 was related to packaging [2], mainly in products with short useful lives, such as bags and bottles. As a result of the growing production and discard, plastic waste has become an issue of global concern, accounting for 12% of the waste produced worldwide [3]. Conventional plastics do not biodegrade quickly, as microbial species that can metabolize polymers are rare in nature [4].

Different strategies have been implemented in order to decrease the environmental impact of plastic waste. While some of them focus on reducing the consumption and use of plastics, such as plastic bag bans, other interventions tackle the end-of-life impacts through recycling and energy recovery [5]. However, the recovery of the material or energetic value of plastics requires complementary actions, such as separate collection or sorting and infrastructures [5,6,7]. The spread of the recycling and recovery of the heat capacity has been limited by a lack of technical or economic resources, especially in developing countries. As a result, a significant proportion of plastics end up buried in landfills and on dumpsites. Besides, improper management leads to losses of plastic waste to the environment [8]; it was calculated that 6.2 Mt of macroplastics were lost to the environment in 2015 [9], and it was estimated that 50–90% of marine debris is plastic [10]. For this trend to change, the current infrastructure for waste management would need to increase by fourfold [11].

In this scenario, biodegradation of plastics has been promoted as an alternative to decreasing these materials’ impact at the end of their useful life. This attribute is expected to be helpful for the plastic products which are most likely to be mismanaged or whose recycling is not feasible [12], such as packaging and medical products [13]. Alternatives include biobased and biodegradable plastics, such as polyhydroxyalkanoates (PHA) [14] and novel materials produced from proteins and polysaccharides [15]. They are considered a promising option to decrease the plastics’ environmental impact by lowering the use of fossil resources and promoting these materials’ integration into natural cycles. Different microorganisms capable of degrading conventional plastics in laboratory conditions have been isolated. The enzymatic process usually happens in two stages: surface adsorption of enzymes and hydroperoxidation or hydrolysis of chemical bonds [16], and is highly dependent on the chemical structure of the plastics [17]. However, the specific process of polymer cleavage by bacteria remains largely unknown [18].

The idea of biodegradable plastics appeals to consumers [19]. However, their potential to be a solution to plastic pollution is uncertain, given that the conditions required for biodegradation are not always fulfilled in the environment [20] or in waste management scenarios. Two main types of plastics have been promoted as biodegradable in developing countries: compostable, which degrade aerobically by the action of microorganisms under the conditions of an industrial composting plant [21,22,23], and oxo-degradable, which degrade in a sequential abiotic-biotic process triggered by the oxidation promoted by UV radiation [24,25,26].

The consumption of plastics in Mexico reached 6221 kTon in 2019 (50 kg/person/year); 3499 kTon were covered by local production, and the rest was imported [27]. As a result of the increase in plastic waste, oxo-degradable and compostable plastics have been promoted by local authorities to substitute conventional plastics, especially for carrying bags. However, in Mexico, as well as in other Latin American countries, compost facilities are scarce. This lack of infrastructure and improper waste management practices by consumers [28] provoked that the most likely end-of-life scenario for plastics sold as biodegradable are landfills and dumpsites, where aeration, microorganism population, and humidity will be remarkably different from those of composting. The need to develop research that realistically predicts the degradability of plastics has been signaled before. Although there were studies focused on the analysis of biodegradation of plastics in anaerobic environments [29], most of them aimed to assess digestion processes [30], which do not represent the conditions found in landfills and dumpsites: long retention times (in the order of decades), lack of aeration and mixing, heterogeneity, varying temperature, and moisture [31]. The goal of our research was to contribute to filling this gap of knowledge. In this context, this research aimed to assess the in-laboratory degradation of compostable, oxo-degradable, and conventional plastics under the burial conditions that could be found in local landfills in order to determine the fate of these materials in the current waste management conditions. Besides comparing the different materials, we tried to assess if they would degrade in the current waste management conditions, as well as the consequences that could be inferred from their performance.

## 2. Materials and Methods

In this research, three different plastic films available in the Mexican market were used to assess their behavior in landfill conditions. Ecovio^®^, a certified compostable plastic produced by BASF, is produced by combining the compostable fossil-based plastic Ecoflex^®^ (polybutylene adipate terephthalate, PBAT) and biobased polylactic acid [32]. High-density polyethylene (HDPE) containing the prooxidant additive d2w^®^ and a conventional HDPE with the same gauge were used as examples of oxo-degradable and conventional plastic films used in the production of plastic bags. Conventional HDPE and oxodegradable HDPE were provided by Plásticos Degradables (Cuernavaca, Mexico), while ECOVIO^®^ was provided by the Mexican filial of BASF.

### 2.1. Preparation of the Solid Waste Mixture

A mixture resembling the composition of the urban solid waste of Mexico City, as reported previously [33], was prepared (Table 1) to guarantee similar characteristics when assessing the plastics. All the materials were cut roughly into 5 cm × 5 cm squares and mixed with a shovel. The mixture was divided into three portions, and each one was amended with a specific type of plastic film: HDPE, oxo-degradable polyethylene (HDPE-OXO), and ECOVIO^®^. In all cases, plastic films, cut into 5 cm × 10 cm strips, accounted for 6.5% (in weight) in their corresponding waste mixture. This proportion reflects the proportion of plastic bags in the waste of the city.

### 2.2. Simulation of Landfill Conditions in the Laboratory

The simulation of landfill conditions was carried out in reactors named lysimeters. Each lysimeter is a cylinder (1.9 m height, 0.5 m diameter) built with acrylic. They have a stainless-steel perforated plate in their base, which works as a support for the waste. Valves located at the base and the top of the lysimeters allowed for leachate extraction and recirculation. A manometer was located at the top to measure the increase in pressure produced through biogas.

Each lysimeter was filled with a waste mixture containing a specific type of plastic film (HDPE, HDPE-OXO, or ECOVIO^®^). Before introducing each waste mixture into its corresponding lysimeter, water was added (52.3% in mass) to reach the field capacity and promote leaching. A 10 cm layer of porous rock was placed in the base, and then the waste was added to form a 1.47 m column, compacted to 418.5 kg/m^3^. A top layer, composed of 0.1 m porous rock and 0.05 m compost, was added to resemble the cover in a landfill cell. A perforated plate was placed at the top to distribute leachate during the recirculation. The systems were tested to ensure no gas leaks were present, and the biogas produced by the biodegradation of the waste was stored in an inflatable vinyl bag. Lysimeters were covered with a black cover to simulate the dark conditions in a landfill.

### 2.3. Monitoring of the Process

Temperature, moisture, and conductivity inside the lysimeters were measured in real-time using a Decagon Devices ECH2O sensor. A manometer at the top allowed to follow the biogas production up. The production and composition of biogas were measured weekly. The biogas composition was measured in a gas chromatograph Agilent Technologies 7890B equipped with an ECD detector, and the amount of biogas produced was measured by the displacement of liquid (Bagi et al., 2007). After the biogas, remotion leachate was recirculated (30% *v*/*v*) to promote biological processes [34]. The monitoring of sampling included pH, measured potentiometrically, and chemical oxygen demand (OCD), assessed with a HACH by the EPA method 8043.

### 2.4. Assessment of the Degradation of Plastics

After 854 days, waste was removed from the lysimeters, and the plastic strips were recovered. They were washed carefully with a 1:50 (*v*/*v*) chloride solution and rinsed with distilled water, to be later dried at room temperature. The plastic strips were visually inspected to look for changes in color, holes, and fissures. Degradation was measured by the loss of mechanical properties by assessing the loss in elongation at break. This was measured in a universal test machine LF Plus following the ASTM D882-12 method. Only complete strips, without holes or fissures, were used for this test, which was performed for ten samples of each plastic. The chemical changes in the plastics were assessed by Fourier Transform Infrared Spectroscopy (FTIR) in a Perkin Elmer Spectrum 2 equipment. This analysis allowed for the measurement of different functional groups produced by degradation. The final molecular weight of the plastics was assessed by gel permeation chromatography in an Agilent PL-GPC 220. Finally, samples of each plastic film were analyzed by scanning electron microscopy using JSM 6610LV equipment.

## 3. Results

The main characteristics of biogas and leachate were analyzed during the 854 days of the experiment. A leak of gas in the reactor containing ECOVIO^®^ prevented a precise measurement of the biogas produced in that lysimeter. However, the positive pressure generated inside that reactor allowed maintaining the anaerobic conditions that developed inside the reactors. The Lysimeter containing ECOVIO^®^ reached its maximum methane concentration around day 280, while for PEAD, it was reached around day 400. This parameter reached a plateau in both cases, which lasted until day 600 before beginning a gradual decrease. The quality of biogas for PEAD-OXO was different, given that the maximum amount of methane was achieved around day 600. The composition of the biogas during the experiment is shown in the Supplementary Materials.

Leachate had an initial acidic condition in the three reactors (pH = 5.2) due to the dissolution of volatile fatty acids produced by organic waste degradation. This condition remained stable during the 200–250 days of latency for biogas production. Afterward, the pH increased until it reached values close to 7.0. Neutrality coincided with the decrease of methane in biogas, as expected. Similar behavior was observed before [35,36]. The DQO showed a constant decrease along with the experiments, from initial values in the 36,000–48,000 mg/L range to end values ranging from 284 mg/L to 1553 mg/L.

### 3.1. Visual Assessment of Degradation

**Figure 1:**
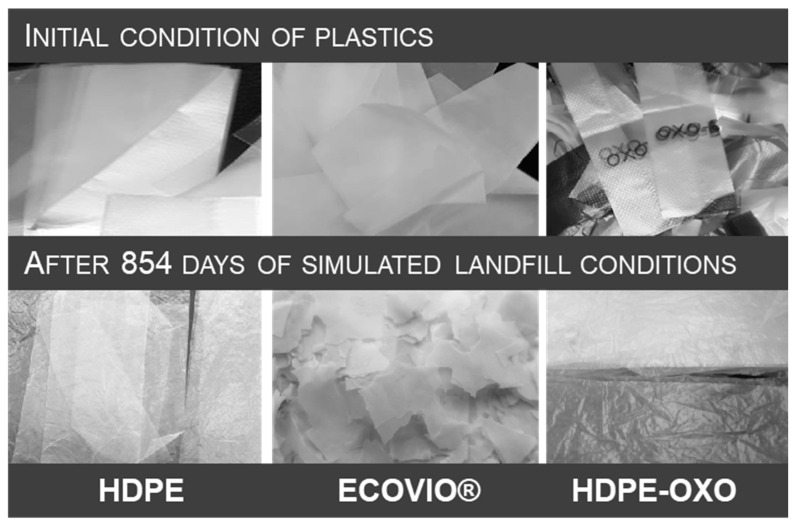
High-density polyethylene, compostable plastic ECOVIO^®^ and oxo-degradable polyethylene films before and after degradation in simulated landfill conditions.

Degradation of plastics commonly requires work with two or more techniques [37], which, combined, were used to assess the extent of both biotic and abiotic processes. The simulation of landfill conditions produced visible effects in the ECOVIO^®^ films, which showed changes in color, embrittlement, and fragmentation (Figure 1). As a result, it was not possible to retrieve complete strips of this plastic. On the other hand, both polyethylenes showed no significant changes in their shape or size.

### 3.2. Loss of Mechanical Properties

**Figure 2:**
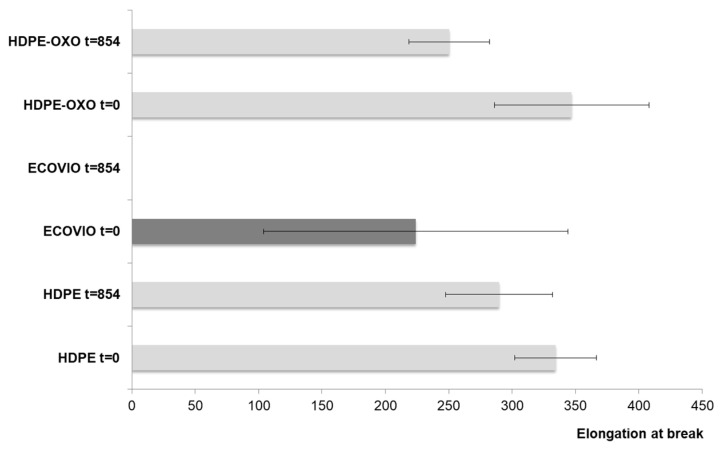
Elongation at break of plastic films before and after degradation. Error bars show the standard deviations of ten samples.

The loss of mechanical properties is used as an indicator of plastic degradation [38,39,40,41]. In this case, the elongation at break of the films was measured at the end of the experiment and compared to their initial value. It was not possible to measure the final value for ECOVIO^®^ because of the fragmentation of this plastic (Figure 1), which indicated a complete loss of physical integrity that could be related to the degradation. While PEAD and PEAD-OXO both showed some loss of the referred property (Figure 2), there is no significant statistical difference between the initial and final values. On average, the elongation at break decreased by 27% for PEAD-OXO and 13% for PEAD; the higher loss of mechanical properties in OXO-PEAD could be expected because of the prooxidant additive in the plastic. The elongation at break is not linearly related to the plastics’ chain length; even an extensively weathered, embrittled plastic has an average molecular weight in the tens of thousands g/mol [4].

### 3.3. Changes in Chemical Composition

**Figure 3:**
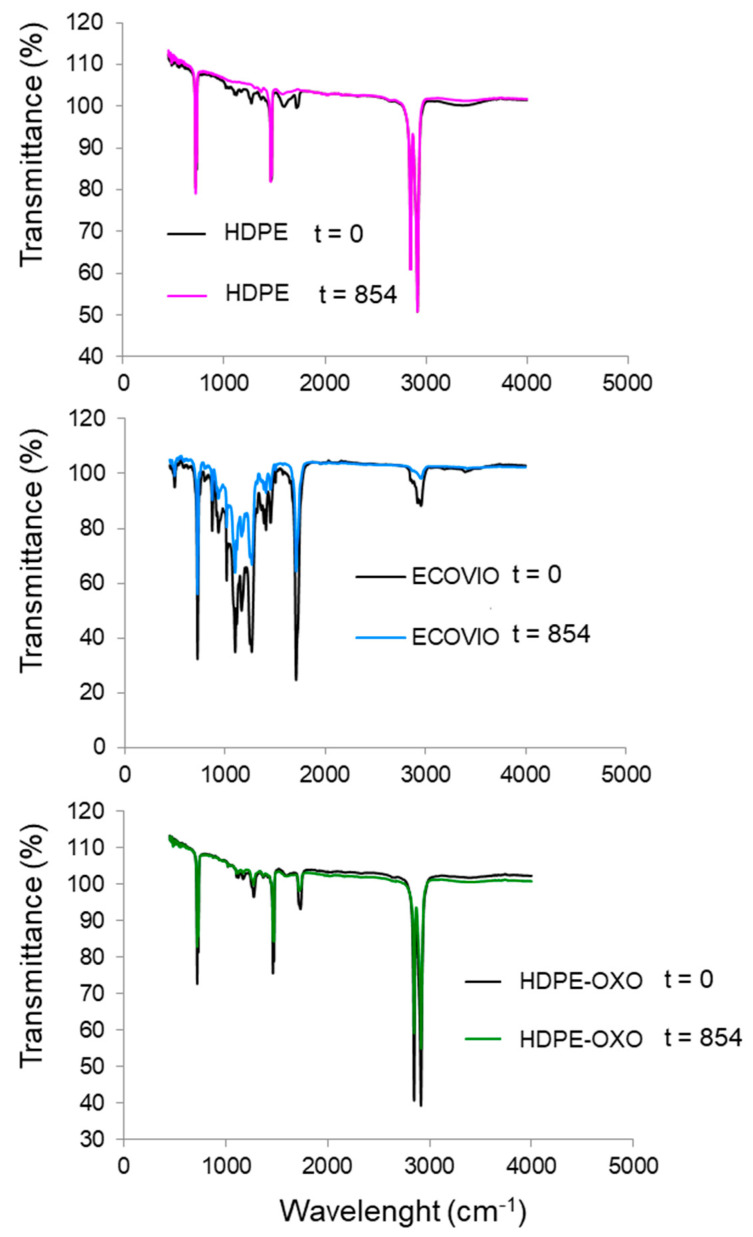
Infrared spectra of high-density polyethylene, compostable plastic ECOVIO^®^, and oxo-degradable polyethylene films before (t = 0) and after degradation (t = 854 days).

FTIR allowed the identification of distinct functional groups based on previously reported data [40,42,43,44,45]. As shown in Figure 3, the conventional HDPE profile did not show significant changes, as it kept the same characteristic peaks [46,47]. A decrease in the peaks related to the C=O and OH groups was also observed. Similar behavior was observed in PEAD-OXO. On the other hand, ECOVIO^®^ showed a decrease in the intensity of the peaks, which was related to the higher mobility of the molecules due to changes in the flexibility of the film [48]. The main change was the disappearance of the peak attributed to the ketonic C=O groups.

### 3.4. Decrease in Molecular Weight and SEM

**Figure 4:**
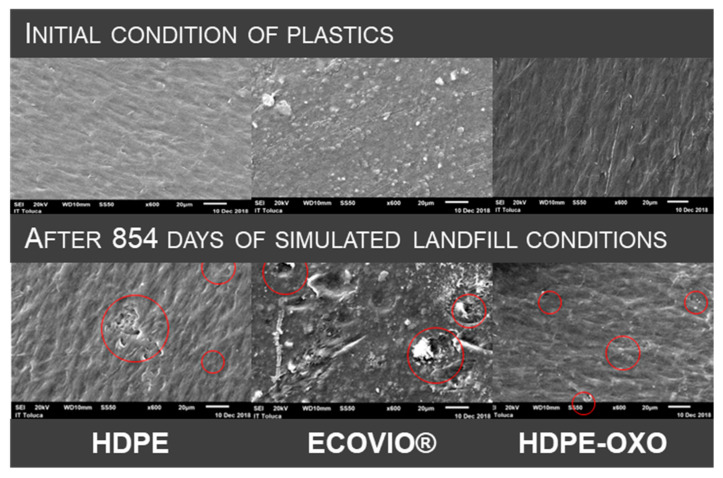
SEM images of high-density polyethylene, compostable plastic ECOVIO^®^, and oxo-degradable polyethylene films after exposition to simulated landfill conditions.

Photodegradation and thermal degradation of plastics can cause changes in the average molecular weight (MW) and MW distribution [49]. This shortening in the molecule length is very relevant and is generally considered the first and necessary step to promote plastic mineralization. It was proposed that polyethylene, oligomers produced by abiotic degradation, could be biodegraded when they reach an MW of 500 g/mol [4]. At the end of the experiment, ECOVIO^®^, HDPE, and HDPE-OXO had a molecular weight of 27,522, 218,460, and 191,545 g/mol, respectively. The SEM images (Figure 4) show the deterioration in the surface of all the plastics. Precipitation, possibly caused by salts, could also be observed. ECOVIO^®^ showed punctures and fissures, while both polyolefins presented only small holes.

## 4. Discussion

Plastic degradation in the environment and different end-of-life scenarios are usually the result of simultaneous or consecutive abiotic and biotic processes. The process is commonly begun by abiotic oxidation or hydrolysis. Oxidation is commonly associated with photo or thermal degradation. It can be hindered by the depletion of oxygen in landfills, decreasing the abiotic processes that would take place in surface weathering conditions. Light-induced oxidation is considered by several orders of magnitude faster than other degradation processes [4]. Hydrolysis is relevant for plastics containing different atoms than carbon in their main chain. It can be enzyme-induced, producing significant loss of mass due to surface erosion of the material, or begin with an abiotic process, which changes properties but does not significantly affect mass loss [50].

Polyethylene showed a low level of degradation in the landfill simulation experiment, as evidenced by a low loss of mechanical properties and minimal chemical structure changes. Previous research found that the degradation of this plastic in landfills was related to the level of oxygen present in the media, which tends to vary with depth [51]. However, evidence showed that, given enough time (i.e., more than 20 years), physical degradation of PE in real landfills was possible, as microplastics (100–1000 µm) were found in leachate [52]. Fragmentation, loss of gloss, and augmentation in carbonyl index were found in samples excavated from landfills [53], suggesting that oxidative processes were the leading cause of the fragmentation process [54].

Low degradation of PE by bacteria and consortia isolated from landfills [42,55,56] and in other anaerobic environments was found before [57]. However, reported results only described partial processes which did not reach mineralization [58]. Additionally, in those experiments, it was common to use a pretreatment to promote the enzymatic attack of the PE. The proposed mechanism included the formation of free radicals from tertiary carbons, with the production of CH_4_ and H_2_ [59]. It was also shown that fungi could produce pits and erosions in plastics [50]. However, it was reported that the apparition in those holes could take decades and measurable weight loss when PE is buried in the soil. The environmental conditions in an oxygen-depleted landfill or dumpsite could increase the time for these processes to take place. Based on this evidence, it can be concluded that the oxygen profile in a landfill will be the main driver for the fragmentation of PE films. Furthermore, it was suggested that plastics could inhibit anaerobic degradation processes; polypropylene microplastics (also a polyolefin) decreased 58% of the methanogenic activity in the anaerobic treatment of wastewater [60]. Further research is needed to clarify if any possible inhibition was caused by chemical or physical characteristics and behavior, such as possible mass transfer interference.

The addition of pro-degradant additives in the oxo-degradable film produced a low increase in the degradation rate of PE, mainly evidenced by a loss of elongation at break, punctures, and holes in the films, which were mainly related to surface degradation, as found before [61]. These surface morphology changes were not directly related to bulk property changes [25]. Although a biofilm formation was found in this type of plastics [26,62,63], the abiotic–biotic process was triggered by weathering. The limitation of UV radiation and oxygen hindered the process in landfills, as photodegradation was the primary mechanism for the abiotic degradation stage [64]. This requirement of an abiotic degradation process, triggered mainly by UV radiation, was one of the main barriers to the degradation of oxo-degradable plastics in landfills and dumpsites, where most of them would be buried in the dark. The use of additives that caused observable changes in color, when the desired abiotic photodegradation was achieved, was suggested as an option to guarantee this technology’s proper performance [64].

Further, the compostable plastic ECOVIO^®^ evidenced higher degradation through the complete loss of physical integrity, decreased C=O groups, and had a low final molecular weight. Aliphatic polyesters such as polyhydroxybutyrate (PHB) and polycaprolactone (PCL) could degrade (measured by weight loss) in strictly anaerobic conditions [65]. Polylactic acid (PLA), one of the components of the compostable plastic tested in this study, was considered less degradable in anaerobic conditions than PHB and PCL [66]. It showed different levels of anaerobic degradation depending on the environmental conditions. It could be biodegraded (91%) in environments such as an anaerobic sludge [67]. Although PLA anaerobically biodegraded and produced a high yield of biogas, the time frame of PLA digestion was much longer than that of biowaste, making it unacceptable on a technical scale under mesophilic conditions [68]. In other studies, PLA degradation on anaerobic conditions was almost neglectable [57].

Reported results for PLA degradation in landfills vary depending on recirculation rates, temperature, and oxygen [69]. Further, the degradation of PBAT (the second component of ECOVIO^®^) by hydrolysis was proved by the imaging analysis and quantification of degradation products in biogas sludge. However, the detected hydrolysis rates were still too low for an efficient PBAT degradation in industrial biogas plants, much slower than food waste [70].

Besides the oxygen profile, the rate of the hydrolytic degradation of compostable plastics in landfill conditions could be affected by UV exposure [71], temperature, pH, and water [25]. As a source of UV radiation, sunlight could promote oxidation and increased wettability and intensity of carbonyl peaks in the three types of plastics assessed in this research [71]. In the case of water, if the diffusion was high in the films, bulk degradation occurred promoting a molecular weight decrease. This phenomenon is typical for polyesters such as PCL and PLA and caused a non-linear mass-loss over time by causing a decrease in Mw, mechanical properties, and mass [72]. As leachate recirculation usually accelerated the decomposition of waste [73,74], it could be expected to increase rates of degradation of compostable plastics in landfills. Medium pH also influenced the hydrolytic degradation mechanism of polyesters, which affected the degradation kinetics [75].

Besides environmental conditions, such as temperature, humidity, sunlight, water, and oxygen availability [64], factors intrinsic to the degradable plastics would affect the extent and rate of degradation. These included their degree of crystallinity [73], thickness [64], C/N ratio [76], presence of additives, and antioxidants [58]. The process would also be influenced by the composition of the waste mixture and the availability of alternative carbon sources, which could complement or compete with plastics [58,76]. Degradable plastics could also affect the global biodegradation of the waste mixture, or the quality of the formed biogas, as shown for PLA [77].

The use of biodegradable plastics has been promoted without proper attention to labeling or communication with society and decision-makers, leading to misunderstandings and false expectations [78]. The concern was not only limited to the feasibility of degradation: landfilling biodegradable plastics could lead to undesirable outcomes, such as an increase in methane production, the complexity of leachate, changes in the temperature profile, and accumulation [79].

## 5. Conclusions

Due to the governments and society’s interest in decreasing plastic waste accumulation in landfills and the environment, different degradable plastics can be found in the market. In emerging economies, it is common that such an introduction lacks a complete regulatory framework that allows consumers to distinguish among the different options and their possible end-of-life impact. In Mexico, for example, the lack of eco-labels and certification schemes provoked that different plastics, including oxo-degradable polyolefins, are promoted as biodegradable. Due to this disinformation and insufficient waste management infrastructure, plastic films’ most common fate is their burial in dumpsites and landfills.

In this research, the assessment of the degradation of conventional and oxo-degradable plastics was carried out in reactors simulating landfill conditions for almost three years. The different parameters used to assess degradation allowed us to conclude that, as expected, HDPE evidenced only a low-level surface erosion and minimal changes in mechanical properties, which increased slightly with the presence of the prooxidant additive. Further, although degrading faster, the compostable film could still be identified in the waste mixture. It might seem obvious, but the promotion of these materials, if they ended up in sites different from a composting plant, was not a solution for the plastic waste problem.

Landfills and dumpsites present different conditions, depending on their location, management, and waste characteristics. Although each site has a particular profile, features such as a continuous depletion of oxygen can be expected. For polyolefins, this condition, added to the lack of UV produced by burial, slowed down the degradation processes as compared with the possible behavior of these plastics when exposed to the surface. The presence and flow of water were also relevant, as they favored the mass transfer and hydrolytic process required for the degradation of compostable plastics. It should be noticed that, in this experiment, periodic recirculation of leachate could have increased the degradation rates. In real sites which do not operate as bioreactor landfills or where leachate recirculation is limited, lower degradation levels could be expected, i.e., the time required to achieve the same level of degradation will be longer.

The attained degradation of the plastics in this assessment resulted from concurrent abiotic and biotic factors, as it was not possible to isolate the biodegradation process. This fact reflects the difficulty of assessing biodegradation when experimental conditions try to reproduce the conditions of real landfills. However, based on the results, it is evident that oxo-degradable and compostable plastics will not biodegrade readily in landfills. This fact should be known and understood for decision-makers to match the materials’ characteristics to the features of the waste management systems.

It is clear that for countries like Mexico, where landfills and dumpsites are the most common destiny for plastic waste, the use of degradable plastics needs to be coupled with regulations and changes in waste management. The expected performance of the different materials available in the market must be identified, in order to couple the used plastics with the available management options that really promote a decrease in environmental impacts. For compostables, certification schemes, ecolabeling, separation at source and during collection, as well as infrastructure are required in order to guarantee the arrival of these plastics at composting plants. For oxo-degradables, more research is required in order to define the optimal conditions for their degradation and to translate them into real operational situations during waste management. If these requirements are not fulfilled and these types of degradable plastics just go to landfills, their use will not produce any tangible environmental benefits.

## Figures and Tables

**Table 1:** Composition of the waste mixture.

Waste	% Weight
Food and yard waste	44
Paper and cardboard	20
Glass	4
Metal	4
Textile	4
Foamed and rigid plastics	6.5
Plastic film	6.5
Wood	1
Disposable diapers	7
Other	5

## Data Availability

The data presented in this study are available in the Supplementary Material.

## Supplementary Materials

The following is available online at https://www.mdpi.com/2073-4360/13/7/1014/s1; Figure S1: Composition of biogas; Figure S2: DQO evolution in leachate.
