# Supplementary figures and images for: Degradation of Plastics in Simulated Landfill Conditions
Source: Polymers (Basel). 2021 Mar 25;13(7):1014. doi: 10.3390/polym13071014 (PMC8037001; doi:10.3390/polym13071014)

## SUPPLEMENTARY MATERIAL

Figure 1. Composition of biogas

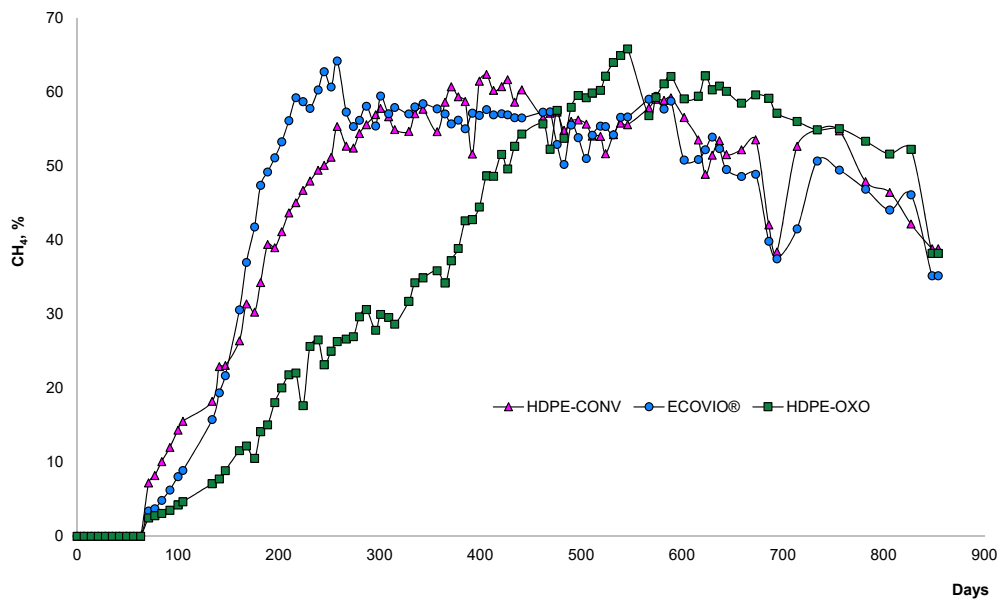

Figure 2. DQO evolution in leachate

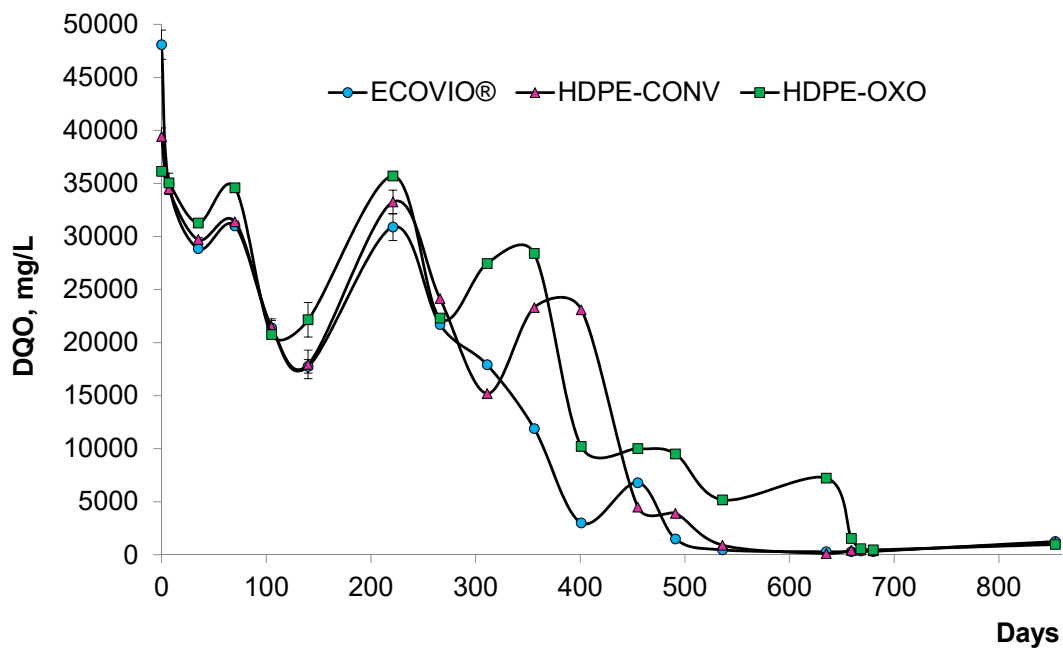

Supplement: Supplementary file 1 [file polymers-13-01014-s001.pdf]
